# A rare case of extraluminal gastrointestinal stromal tumor of the ileum presenting with lower urinary tract symptoms: A case report

**DOI:** 10.1097/MD.0000000000018103

**Published:** 2019-12-10

**Authors:** Chung-Hao Yu, Hui-Kung Ting, Chien-Chang Kao, Wen-Chiuan Tsai, Sheng-Tang Wu, Dah-Shyong Yu

**Affiliations:** a Division of Urology, Department of Surgery, Tri-Service General Hospital, National Defense Medical Center; b Department of Pathology, Tri-Service General Hospital, Taipei, Taiwan.

**Keywords:** differential diagnosis, gastrointestinal stromal tumors, LUTS

## Abstract

**Introduction:**

Benign prostatic hyperplasia, bladder outlet obstruction, and overactive bladder are major causes of lower urinary tract symptoms (LUTS). Tumor compression of the urinary bladder resulting in LUTS was clinically observed. Gastrointestinal stromal tumors (GISTs) presenting with LUTS have not been reported before. Herein, we report a patient with extraluminal GIST of the ileum who had LUTS without gastrointestinal symptoms during the clinical course.

**Patient concerns:**

A 68-year-old man visited the genitourinary outpatient department because of frequent urination with mild dysuria. He also complained of poor appetite, fatigue, and body weight loss of 10 kg over 6 months. A large presacral solid mass lesion compressing the bladder and surrounded by the bowel with gas content was identified through abdominal computed tomography.

**Diagnosis:**

GIST of the ileum with mesenteric invasion was revealed by pathological examination.

**Interventions:**

Exploratory laparotomy with removal of the pelvic tumor and segmental resection of the ileum was performed.

**Outcomes:**

Now, he received adjuvant imatinib target therapy for 1 year with stable condition.

**Conclusion:**

Extravesical compression or invasion of the urinary bladder by a pelvic mass lesion is common but is rarely accompanied by GISTs of the ileum. Specific findings identified through imaging should alert the surgeon to this specific entity and prepare thoroughly before surgical intervention.

## 1 Introduction

Lower urinary tract symptoms (LUTS) generally refer to a combination of urinary symptoms, such as frequency, urgency, and nocturia, relating to storage or voiding disturbances common among older men. Benign prostatic hyperplasia and bladder outlet obstruction are the commonest causes of LUTS.^[1]^ Additionally, overactive bladder may be observed in some men.

During the 1990s, nonepithelial tumors of the gastrointestinal tract were called “gastrointestinal stromal tumors” (GISTs). They originate from the interstitial cells of Cajal and constitute less than 1% of all gastrointestinal tumors. GISTs are most commonly located in the stomach (40%–60%) and jejunum/ileum (25%–30%). The duodenum (5%), colorectum (5%–15%), and esophagus (≤1%) are less common sites.^[2–5]^

Up to 75% of GISTs are discovered when they are less than 4 cm in diameter and are either asymptomatic or associated with nonspecific symptoms. Patients may complain of nonspecific abdominal pain or discomfort, a sensation of abdominal fullness, palpable abdominal mass, malaise, fatigue, or exertional dyspnea with significant blood loss or focal or widespread signs of peritonitis due to perforation.^[2–5]^

## 2 Case report

### 2.1 Patient information

**Figure 1:**
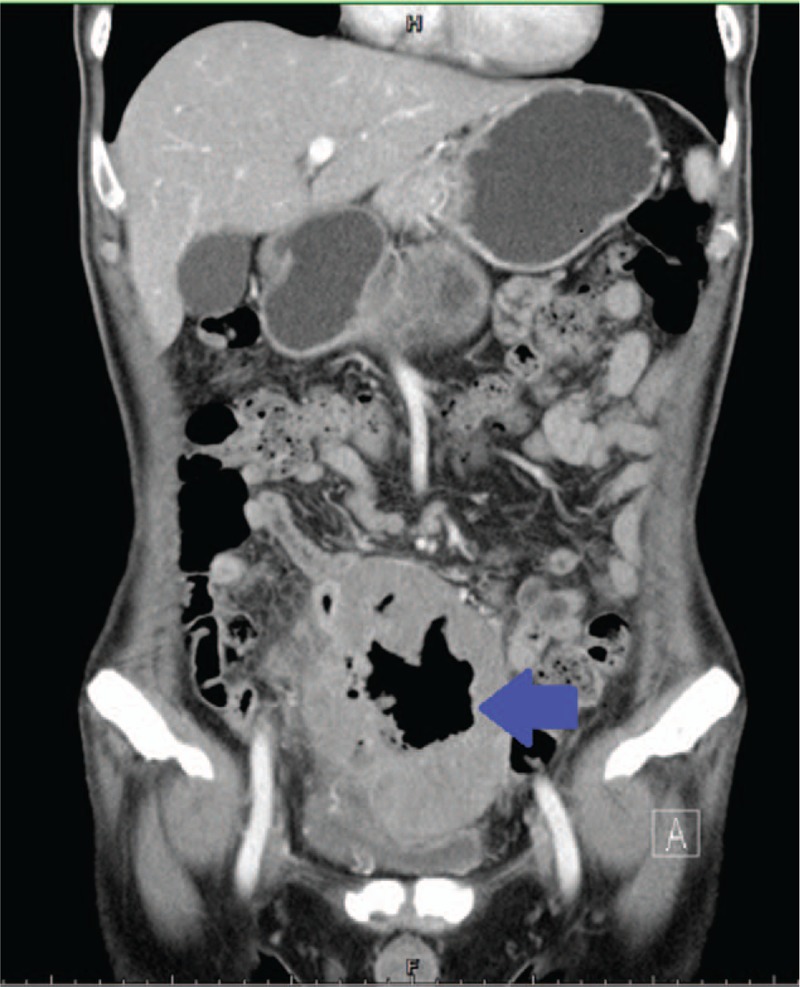
A large, heterogeneous, presacral-located, solid mass (13 × 11 × 9 cm) with gas content, locating posteriorly to the urinary bladder (arrow).

This report presents a 68-year-old man who initially had experienced poor appetite, fatigue, occasional mild lower abdominal discomfort, and body weight loss of 10 kg over 6 months but no nausea, vomiting, diarrhea, or tarry or bloody stool symptoms occurred. Afterward, he noticed frequent urination with mild dysuria. Therefore, he visited the outpatient department for further management in our hospital. Cystoscopy was performed, and the findings were normal in addition to external compression of dome and posterior wall of bladder was seen. Abdominal sonography revealed a solid mass nearby the bladder. Computed tomography (CT) of the abdomen revealed a large, presacral, solid mass lesion (13 × 11 × 9 cm) surrounded by the bowel with gas content that compressed anteriorly against the bladder (Fig. 1).

### 2.2 Surgical and pathological findings

**Figure 2:**
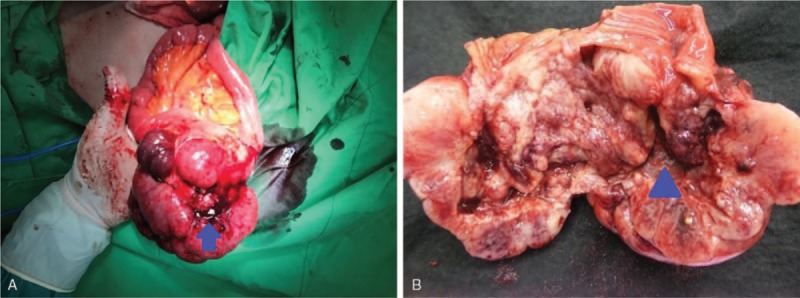
An exophytic solid mass (arrow head) originated from the ileum with fistula tract (arrow).

**Figure 3:**
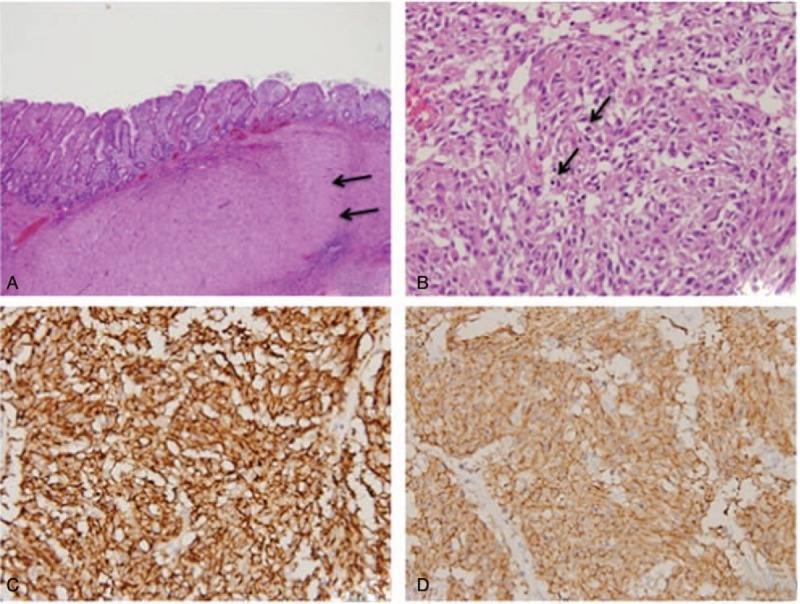
(A) An ill-defined tumor located at the submucosa and muscle layers (arrow) of ileum (H&E, 100×). (B) Hypercellular tumor cells composed of frequent mitotic figures (arrow) (H&E, 400×). Positive immunohistochemical stain of (C) Dog-1 and (D) CD117 (400×).

Exploratory laparotomy with removal of the pelvic tumor and segmental resection of the ileum was performed. A fistula tract from the ileum lumen into tumor was seen in the specimen (Fig. 2). No severe adhesion with easy division between the tumor mass and bladder were observed during operation. The pathological examination revealed that the tumor had invaded the submucosa and muscle layer of the ileum and mesentery (Fig. 3). Dog-1 and CD117 staining were positive according to the immunohistochemistry report. An exophytic GIST of the ileum with fistula formation and mesenteric invasion, pT4N0M0, stage III-B was diagnosed.

### 2.3 Follow-up and outcomes

The patient was referred to the oncology department for 1-year adjuvant imatinib targeted therapy and no tumor recurrence has been noted to date.

## 3 Discussion

Some abdominal masses may compress against the urinary bladder and lead to secondary LUTS, including uterine and rectal masses. Nevertheless, some case reports have revealed that extra-bowel GISTs of the prostate and urinary bladder are associated with urinary tract symptoms.^[2–5]^ However, no patients with GISTs in the ileum with urinary bladder compression and LUTS occurred had been reported.

In such cases, intravenous urography, cystoscopy, or urine cytology can be performed initially; subsequently, CT or MRI may be considered, since GIST screening and staging must be performed through CT or MRI.^[6,7]^ Fluorodeoxyglucose positron emission tomography (PET) is highly sensitive at detecting GISTs and their metastases, since patients with LUTS but no signs of gastrointestinal obstruction are frequently misdiagnosed.

Preoperative biopsy is not usually recommended for resectable lesions that display a high suspicion of GISTs.^[7]^ Primary tumor resection should be performed through standard management and the diagnosis should be confirmed by pathology. Complete resection is possible in most cases of localized GISTs; however, only approximately half of cases remain recurrence free for 5 years or more with surgery alone.

Approximately, 80% of GISTs have a mutation of the KIT protooncogene, a member of the receptor tyrosine kinase family^[8–11]^ and CD117 and Dog-1 are highly specific for GIST.^[12]^

The GISTs were treated using adjuvant and neoadjuvant small molecule tyrosine kinase inhibitors such as imatinib. Following imatinib introduction, the median survival of patients with advanced GIST was 60 months.^[13,14]^ In this study, the patient underwent imatinib treatment for 1 year without complications.

LUTS occurred by ileal GIST tumor compression of bladder is very rare. Clinicians should keep in mind the possible existence of GIST and select appropriate procedures or imaging for diagnosis and proper treatment.

## Author contributions

**Conceptualization:** Sheng-Tang Wu.

**Data curation:** Hui-Kung Ting, Chien-Chang Kao, Wen-Chiuan Tsai.

**Supervision:** Sheng-Tang Wu.

**Writing – original draft:** Chung-Hao Yu, Dah-Shyong Yu.

**Writing – review & editing:** Chung-Hao Yu.
